# Afatinib in EGFR TKI-Naïve Patients with Locally Advanced or Metastatic EGFR Mutation-Positive Non-Small Cell Lung Cancer: A Pooled Analysis of Three Phase IIIb Studies

**DOI:** 10.3389/fonc.2021.709877

**Published:** 2021-07-09

**Authors:** Antonio Passaro, Filippo de Marinis, Hai-Yan Tu, Konstantin K. Laktionov, Jifeng Feng, Artem Poltoratskiy, Jun Zhao, Eng Huat Tan, Maya Gottfried, Victor Lee, Dariusz Kowalski, Cheng Ta Yang, BJ Srinivasa, Laura Clementi, Tejaswini Jalikop, Dennis Chin Lun Huang, Agnieszka Cseh, Keunchil Park, Yi-Long Wu

**Affiliations:** ^1^ Division of Thoracic Oncology, IEO, European Institute of Oncology IRCCS, Milan, Italy; ^2^ Guangdong Lung Cancer Institute, Guangdong Provincial People’s Hospital and Guangdong Academy of Medical Sciences, Guangzhou, China; ^3^ Russian Academy of Medical Sciences, Moscow, Russia; ^4^ Jiangsu Provincial Tumor Hospital, Nanjing, China; ^5^ Petrov Research Institute of Oncology, St Petersburg, Russia; ^6^ Peking University Cancer Hospital & Institute, Beijing, China; ^7^ National Cancer Centre, Singapore, Singapore; ^8^ Tel Aviv University, Tel Aviv, Israel; ^9^ Department of Clinical Oncology, Queen Mary Hospital, The University of Hong Kong, Hong Kong, China; ^10^ Maria Sklodowska-Curie National Research Institute of Oncology, Warsaw, Poland; ^11^ Chang Gung Memorial Hospital, Guishan, Taoyuan, Taiwan; ^12^ HCG Hospital, Bangalore, India; ^13^ Boehringer Ingelheim Italia S.p.A., Milan, Italy; ^14^ Syneos Health, Raleigh, NC, United States; ^15^ Boehringer Ingelheim Taiwan Limited, Taipei, Taiwan; ^16^ Boehringer Ingelheim International GmbH, Ingelheim am Rhein, Germany; ^17^ Division of Hematology Oncology, Samsung Medical Center Sungkyunkwan University School of Medicine, Seoul, South Korea

**Keywords:** afatinib, real world, safety, *EGFR* mutation, EGFR TKI-naïve, NSCLC

## Abstract

**Background:**

Afatinib is approved for first-line treatment of patients with epidermal growth factor receptor mutation-positive (*EGFR*m+) non-small-cell lung cancer (NSCLC). Here, we report findings from a combined analysis of three phase IIIb studies of afatinib in EGFR tyrosine kinase inhibitor (TKI)-naïve patients.

**Methods:**

EGFR-TKI-naïve patients with *EGFR*m+ NSCLC received afatinib 40 mg/day. Dose reductions were permitted for adverse events (AEs). Efficacy endpoints included progression-free survival (PFS), time to symptomatic progression (TTSP), and tumor response. Subgroup analyses were performed by Eastern Cooperative Oncology Group performance status (ECOG PS), presence of brain metastasis, age and common/uncommon *EGFR* mutations (plus other factors).

**Results:**

1108 patients were treated. Median age was 61 years (range, 25–89); 19.2% had baseline brain metastases, 4.4% had ECOG PS ≥2, and 17.9% had tumors harboring uncommon mutations. Treatment-related AEs (TRAEs) were reported in 97.2%, most commonly diarrhea and rash. 41.6% had AEs leading to dose reduction. Median PFS was 13.0 months [95% confidence interval (CI): 12.0–13.8]; median TTSP was 14.8 months (95% CI: 13.9–16.1). Objective response rate (ORR) was 55.0%. Age, presence of baseline brain metastases, major (G719X, L861Q, S768I) or compound uncommon mutations had little/no effect on PFS, TTSP, or ORR, while outcomes were poorer in patients with ECOG PS 2 or exon 20 insertion/T790M mutations.

**Conclusions:**

Afatinib was tolerable with no new safety signals. Afatinib demonstrated encouraging efficacy in a broad patient population, including those with brain metastases or uncommon *EGFR* mutations.

## Introduction

Activating mutations in the epidermal growth factor receptor (*EGFR*) gene, leading to aberrant EGFR signaling, render non-small cell lung cancer (NSCLC) tumors highly sensitive to targeted treatment with EGFR tyrosine kinase inhibitors (TKIs) (1). Based on seminal randomized controlled trials (RCTs) (1), EGFR TKIs are the first-line treatment of choice in patients with advanced *EGFR* mutation-positive (EGFRm+) NSCLC, with five TKIs currently approved. These are: the first-generation reversible EGFR TKIs, gefitinib and erlotinib; the second-generation irreversible ErbB family blockers, afatinib and dacomitinib; and the third-generation irreversible EGFR TKI, osimertinib (2–5).

As an ErbB family blocker, afatinib inhibits signaling *via* all hetero- and homodimers formed by ErbB1 (EGFR), ErbB2 [human epidermal growth factor receptor 2 (HER2)], ErbB3 (HER3), and ErbB4 (HER4) (6, 7). In RCTs, afatinib significantly improved progression-free survival (PFS) versus standard chemotherapy (8, 9). Furthermore, in the LUX-Lung 3 and 6 trials, afatinib significantly improved overall survival (OS) *versus* chemotherapy in patients with tumors harboring Del19 mutations (10). In LUX-Lung 7, afatinib conferred statistically significant improvement in PFS (although there was minimal difference in medians) and time-to-treatment failure versus gefitinib (11). There was no significant difference in OS (12). Across these RCTs, afatinib was tolerable, with few treatment discontinuations due to toxicity. Treatment-related adverse events (TRAEs) were managed effectively with tolerability-guided dose reductions.

RCTs are conducted under highly controlled settings, often with strict inclusion criteria. Consequently, certain patient subgroups are generally under-represented in clinical trials, such as the very elderly and patients with brain metastases, uncommon mutations, prior chemotherapy treatment, or Eastern Cooperative Oncology Group performance status (ECOG PS) ≥2. Accordingly, the importance of assessing the efficacy and tolerability of recently developed drugs in ‘real world’ settings is becoming increasingly recognized (13). To date, available real-world evidence suggests that afatinib is effective and tolerable in diverse patient populations treated in routine clinical practice (14–18). Here, in order to assess outcomes in a larger cohort, we report a combined analysis of three phase IIIb studies of afatinib in EGFR TKI-naïve patients with *EGFR*m+ NSCLC treated in a setting similar to daily clinical practice (19, 20).

## Methods

### Study Designs

Study 1200.55 (NCT01853826; conducted in Europe, Australia, Russia, and Israel), Study 1200.66 (NCT01953913; conducted in Asia), and Study 1200.193 (NCT01931306; conducted in South Korea) were all phase IIIb, open-label, multicenter, single-arm trials of afatinib in EGFR TKI-naïve patients with locally advanced or metastatic *EGFR*m+ NSCLC (**Supplementary Figure 1**). All studies were approved by the Institutional Review Board or Independent Ethics Committee of each participating center, and were carried out in accordance with the Declaration of Helsinki, the International Conference on Harmonisation of Technical Requirements for Pharmaceuticals for Human Use, Good Clinical Practice, and local laws. All patients provided written, informed consent.

### Patients and Treatment

Patients were aged ≥18 years with histologically-confirmed, locally advanced or metastatic *EGFR*m+ NSCLC, adequate organ function, and an ECOG PS of 0–2. Exclusion criteria included: previous use of an EGFR TKI; use of any anti-cancer treatment (or hormonal anti-cancer treatment for Study 1200.193) <2 weeks, radiotherapy (except palliative) <14 days (or <4 weeks for Study 1200.66), and major surgery <4 weeks before the first dose of afatinib; history or presence of cardiovascular abnormalities; pre-existing interstitial lung disease; and symptomatic brain metastases.

Patients received afatinib (starting dose 40 mg once daily) until disease progression, lack of tolerability or other reasons necessitating withdrawal. Investigators could continue afatinib beyond radiological progression for as long as they judged that the patient was benefiting. TRAEs were managed using tolerability-guided dose modifications. In the event of any drug-related grade ≥3 AE, persistent grade 2 diarrhea, or grade ≥2 renal dysfunction, treatment was paused until the severity recovered to grade ≤1 or baseline severity. Treatment could then be resumed at a lower dose (reduced by 10 mg decrements) to a minimum of 20 mg/day. If the patient could not tolerate 20 mg/day, or the patient did not recover to grade ≤1 or baseline within 6 weeks, treatment was discontinued.

### Endpoints and Assessments

The primary objective of each study was to evaluate the safety of afatinib; the secondary objective was to assess the efficacy of afatinib. AEs were graded using the National Cancer Institute Common Terminology Criteria for Adverse Events version 3.0. Efficacy endpoints were chosen to reflect real-world clinical practice and current treatment guidelines, and included: PFS (defined as time from first administration of afatinib to the date of progression or to the date of death, whichever occurred first); time to symptomatic progression (TTSP; defined as the time from first administration of afatinib to the date of first documented clinically significant symptomatic progression); and tumor response. Efficacy analyses were based on the assessment of cancer-related symptoms and, if available, radiologic assessments as per standard of care at the participating institution and determined by Response Evaluation Criteria in Solid Tumors (RECIST). Tumor assessments, and the version of RECIST criteria used in the three studies were undertaken according to local standard of care at each participating site. PFS and ORR were judged by investigator. *EGFR* mutations were detected according to the methodology used at each participating institution.

### Statistical Analyses

All patients who received ≥1 dose of afatinib (treated set) were included in the safety and efficacy analyses. Subgroup analyses were conducted according to: *EGFR* mutation status (common/uncommon); presence of brain metastases at baseline (yes/no); age (<65 years/≥65 years and <75 years/≥75 years); ECOG PS (0–1/2); and line of therapy (first/second/>second). Patients with tumors harboring uncommon *EGFR* mutations were further subdivided into the following five groups: 1) T790M; 2) exon 20 insertions; 3) ‘major’ uncommon mutations (G719X, L861Q, and S768I, with or without any other mutation except T790M or exon 20 insertion); 4) compound mutations; and 5) other uncommon mutations. Outcomes were also assessed for compound mutations including major mutations. Descriptive statistics are presented; no hypotheses testing was planned, and all analyses were exploratory.

## Results

### Patients, Disposition, and Treatment Exposure

Of the 1163 patients enrolled, 1109 entered and 1108 had been treated with afatinib (**Supplementary Figure 1**). Overall, 1081 (97.6%) patients discontinued treatment, the most common reason being progressive disease, in 739 (66.7%) patients. Median age was 61 years (range, 25–89), 38.2% of patients were aged ≥65 years, with 10.7% aged ≥75 years. Most patients (58.3%) were female and were predominantly either Asian (57.7%) or white (42.0%; **Table 1**). An ECOG PS of 2 was reported in 49 (4.4%) patients, and 213 (19.2%) patients had brain metastases. The most common histological classification was adenocarcinoma, in 95.8% of patients.

**Table 1:** Baseline demographics and disease characteristics in the treated set.

Characteristic	Afatinib (n = 1108)
Sex, n (%)	
Female	646 (58.3)
Median age, years (range)	61 (25–89)
≥65 years, n (%)	423 (38.2)
≥75 years, n (%)	119 (10.7)
Race, n (%)	
Asian	639 (57.7)
White	465 (42.0)
Other^†^	4 (0.4)
Smoking status, n (%)	
Never smoked	735 (66.3)
Ex-smoker	307 (27.7)
Current smoker	66 (6.0)
Histological classification, n (%)	
Predominantly adenocarcinoma	1061 (95.8)
Predominantly squamous cell carcinoma	20 (1.8)
Large cell/undifferentiated carcinoma	11 (1.0)
NOS/missing	16 (1.4)
Prior therapy	
Any	578 (52.2)
Chemotherapy/other systemic therapy	373 (33.7)
Radiotherapy	213 (19.2)
Surgery	278 (25.1)
*EGFR* mutation, n (%)	
Common only (del19 and/or L858R)	909 (82.0)
Del 19	556 (50.2)
L858R	429 (38.7)
Uncommon only	198 (17.9)
Missing	1 (0.1)
Baseline ECOG PS, n (%)	
0	285 (25.7)
1	773 (69.8)
2	49 (4.4)
Missing	1 (0.1)
Baseline brain metastases,^‡^ n (%)	213 (19.2)
Prior systemic chemotherapy, n (%)	367 (33.1)

ECOG PS, Eastern Cooperative Oncology Group performance status; EGFR, epidermal growth factor receptor; NOS, not otherwise specified. ^†^Other: one Native Hawaiian or other Pacific Islander; three Black/African American. ^‡^Asymptomatic.

In total, 909 (82.0%) patients had tumors harboring common *EGFR* mutations, while 198 (17.9%) had tumors harboring uncommon mutations only; the most frequent uncommon *EGFR* mutations were insertions in exon 20, which were detected in 70 patients (6.3% overall). Nearly a third of patients (33.1%) had previously received systemic chemotherapy. The median duration of treatment across all lines of afatinib was 12.7 months (range, 0.07–56.1 months). Dose reductions from 40 mg/day to 30 mg/day were performed in 462 (41.7%) patients, 145 of whom (13.1% overall) had a further dose reduction to 20 mg/day.

### Safety

Most patients (1100; 99.3%) experienced an AE, and 620 (56.0%) patients experienced grade ≥3 AEs (**Table 2**). Any-grade TRAEs were reported in 1077 (97.2%) patients, and grade ≥3 TRAEs were reported in 412 (37.2%) patients. The most common TRAEs (any grade/grade ≥3) were diarrhea (89.1%/14.0%), rash (61.6%/9.1%), and paronychia (39.7%/3.6%; **Table 2**). Serious AEs (SAEs) were reported in 403 (36.4%) patients, the most common being malignant neoplasm progression in 53 (4.8%) patients, and pleural effusion in 38 (3.4%) patients; 81 (7.3%) patients had a treatment-related SAE, the most common being diarrhea in 28 (2.5%) patients. AEs leading to dose reduction of afatinib were reported in 461 (41.6%) of patients. The most common reasons for dose reduction were diarrhea in 199 (18%) patients, and rash in 108 (9.7%) patients. AEs leading to discontinuation of afatinib were reported in 160 (14.4%) patients, among whom 58 (5.2%) patients experienced TRAEs leading to drug discontinuation; the most frequent of these was diarrhea in 17 patients (1.5%). A total of 122 patients (11.0%) had an AE that led to death, including malignant neoplasm progression in 41 (3.7%) patients, and respiratory failure in 14 (1.3%) patients. There were five TRAEs resulting in death (decreased appetite, dyspnea, pneumonitis, respiratory failure, intestinal infarction).

**Table 2:** Overall summary of AEs, and most common TRAEs (occurring in ≥10% of patients).

AE, n (%)	Treated set (n = 1108)	
Any AE	1100 (99.3)	
Any grade ≥3 AE	620 (56.0)	
Any TRAE	1077 (97.2)	
Any grade ≥3 TRAE	412 (37.2)	
Any SAE	403 (36.4)	
AEs leading to dose reduction	461 (41.6)	
AEs leading to discontinuation	160 (14.4)	
TRAEs leading to discontinuation	58 (5.2)	
AEs leading to death	122 (11.0)	
Most common TRAEs	All grades	Grade ≥3
Diarrhea	987 (89.1)	155 (14.0)
Rash	683 (61.6)	101 (9.1)
Paronychia	440 (39.7)	40 (3.6)
Stomatitis	243 (21.9)	27 (2.4)
Mucosal inflammation	170 (15.3)	20 (1.8)
Mouth ulceration	149 (13.4)	10 (0.9)
Dry skin	144 (13.0)	2 (0.2)
Pruritus	135 (12.2)	3 (0.3)

AE, adverse event; SAE, serious adverse event; TRAE, treatment-related adverse event.

### Efficacy

#### PFS

**Figure 1:**
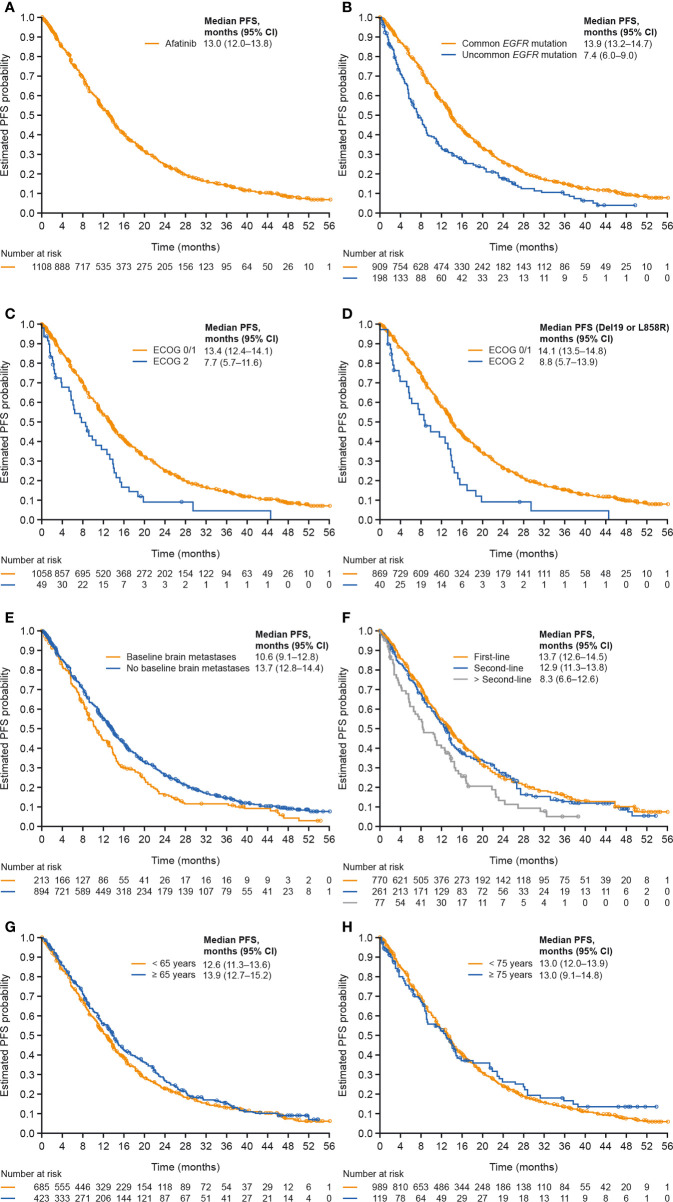
PFS in **(A)** all patients, **(B)** patients with tumors harboring common versus uncommon mutations, **(C)** patients with ECOG PS 0/1 versus 2, **(D)** patients with common mutations and ECOG PS 0/1 versus 2, **(E)** patients with versus without baseline brain metastases, **(F)** patients treated with afatinib in first, second and later lines of therapy, **(G)** patients aged <65 or ≥65 years, and **(H)** patients aged <75 or ≥75 years. CI, confidence interval; ECOG PS, Eastern Cooperative Oncology Group performance status; PFS, progression-free survival.

Median PFS was 13.0 months overall and was 13.9 months among patients with tumors harboring common mutations (**Table 3**; **Figures 1A, B**). Median PFS was longer in patients with ECOG PS 0/1 compared to those with ECOG PS 2 (median: 13.4 and 7.7 months, respectively), and this was also the case among only those patients with tumors harboring common mutations (median, 14.1 and 8.8 months; **Table 3**; **Figures 1C, D**). Median PFS was slightly longer in patients without compared to those with brain metastases at baseline (median, 13.7 and 10.6 months; **Figure 1E**), and in patients treated with first-line afatinib compared to second- or later-line afatinib (median, 13.7, 12.9 and 8.3 months, respectively; **Figure 1F**), while age had little or no effect on PFS (**Table 3**; **Figures 1G, H**).

**Table 3:** Post-hoc analysis of TTSP and PFS for specified subgroups.

Category	Patient subgroup			
All patients				
N	1108			
Median PFS, months (95% CI)	13.0 (12.0–13.8)			
Median TTSP, months (95% CI)	14.8 (13.9–16.1)			
*EGFR* mutation type^†^	Common^†^		Uncommon^‡^	
N	909		198	
Median PFS, months (95% CI)	13.9 (13.2–14.7)		7.4 (6.0–9.0)	
Median TTSP, months (95% CI)	16.1 (14.8–17.7)		8.3 (7.2–11.0)	
Common mutation type	Del19		L858R	
N	531		378	
Median PFS, months (95% CI)	14.5 (13.8–15.9)		12.6 (11.1–13.8)	
Median TTSP, months (95% CI)	17.2 (15.5–19.3)		14.5 (13.1–16.5)	
ECOG PS	0/1		2	
N	1058		49	
Median PFS, months (95% CI)	13.4 (12.4–14.1)		7.7 (5.7–11.6)	
Median TTSP, months (95% CI)	15.2 (14.1–16.6)		9.9 (7.6–13.9)	
ECOG PS (patients with common mutations)^†^	0/1		2	
N	869		40	
Median PFS, months (95% CI)	14.1 (13.5–14.8)		8.8 (5.7–13.9)	
Median TTSP, months (95% CI)	16.6 (15.1–18.1)		9.9 (7.6–14.5)	
Afatinib line of therapy	First-line	Second-line		>Second-line
N	770	261		77
Median PFS, months (95% CI)	13.7 (12.6–14.5)	12.9 (11.3–13.8)		8.3 (6.6–12.6)
Median TTSP, months (95% CI)	16.0 (14.4–17.7)	13.8 (12.7–15.4)		10.6 (7.6–14.8)
Brain metastases at screening^§^	Yes		No	
N	213		894	
Median PFS, months (95% CI)	10.6 (9.1–12.8)		13.7 (12.8–14.4)	
Median TTSP, months (95% CI)	13.7 (11.0–14.8)		15.5 (14.1–16.9)	
Age, years	<75 years		≥75 years	
N	989		119	
Median PFS, months (95% CI)	13.0 (12.0–13.9)		13.0 (9.1–14.8)	
Median TTSP, months (95% CI)	14.8 (13.8–16.1)		14.8 (13.1–22.3)	
Age, years	<65 years		≥65 years	
N	685		423	
Median PFS, months (95% CI)	12.6 (11.3–13.6)		13.9 (12.7–15.2)	
Median TTSP, months (95% CI)	13.8 (12.9–15.1)		17.5 (15.0–20.6)	

CI, confidence interval; ECOG PS, Eastern Cooperative Oncology Group performance status; EGFR, epidermal growth factor receptor; NE, not evaluable; PFS, progression-free survival; TTSP, time to symptomatic progression. ^†^Patients with EGFR mutation categories of Del19 only or L858R only. ^‡^Patients with EGFR mutation categories other than Exon19 only and L858R only. ^§^Asymptomatic.

#### TTSP

Median TTSP was 14.8 months overall and was 16.1 months in patients with tumors harboring common mutations (**Table 3**; **Supplementary Figures 2A, B**). Median TTSP was numerically longer in patients with ECOG PS 0/1 *versus* 2 (median, 15.2 and 9.9 months) including among only those with common mutations (median, 16.6 and 9.9 months; **Table 3**; **Supplementary Figures 2C, D**). Median TTSP was slightly longer in patients without baseline brain metastases compared to those with brain metastases at baseline (median, 15.5 and 13.7 months; **Supplementary Figure 1E**), and in patients treated with afatinib in first line compared with second or later lines (median, 16.0, 13.8 and 10.6 months, respectively; **Supplementary Figure 2F**). Age had little or no effect on TTSP (**Table 3** and **Supplementary Figures 2G, H**).

#### Tumor Response

Overall, 609 of the 1108 treated patients (55.0%) had an objective response, including 40 (3.6%) complete responses and 569 (51.4%) partial responses. An additional 368 (33.2%) patients had stable disease, for a disease control rate of 88.2% (n=977). Median duration of objective response (DOR) in the overall treated set was 13.2 months (95% CI: 12.2–14.4), and median duration of disease control was 14.1 months (95% CI: 13.6–14.8; **Supplementary Table 1**).

#### Patients with Uncommon Mutations

**Figure 2:**
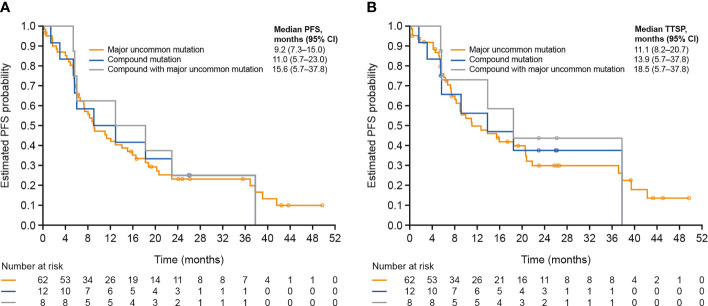
**(A)** PFS and **(B)** TTSP according to type of uncommon *EGFR* mutation. CI, confidence interval; EGFR, epidermal growth factor receptor; PFS, progression-free survival; TTSP, time to symptomatic progression.

Baseline characteristics of patients with uncommon mutations were generally consistent with the overall treated set (**Table 4**). Compared with the T790M and exon 20 mutation subgroups (median PFS, 3.9 and 5.6 months, respectively), median PFS was longer in the compound, ‘major’ and ‘other’ mutation subgroups (11.0, 9.2, and 8.6 months, respectively), particularly in the subgroup with compound mutations with a ‘major’ uncommon mutation (15.6 months; **Figure 2A**). Median TTSP was also longest in the ‘compound with major mutation’ subgroup (18.5 months), followed by the compound mutation (13.9 months), ‘major’ mutation (11.1 months), ‘other’ mutation (9.7 months), exon 20 mutation (5.9 months), and T790M (3.8 months) subgroups (**Figure 2B**). Objective response rates were higher in the compound/’compound with major’, and ‘major’ uncommon mutation subgroups compared with the exon 20 mutation and T790M subgroups, as was the corresponding DOR (**Supplementary Table 2**).

**Table 4:** Baseline demographics and disease characteristics according to the type of uncommon *EGFR* mutation.

Characteristic	T790M (n = 8)	Exon 20 (n = 36)	Major (n = 62)	Compound (n = 12)	Compound with major (n = 8)	Other (n = 5)
Sex, n (%)						
Female	3 (37.5)	22 (61.1)	31 (50.0)	8 (66.7)	5 (62.5)	4 (80.0)
Race, n (%)						
Asian	1 (12.5)	3 (8.3)	41 (66.1)	8 (66.7)	6 (75.0)	0
White	7 (87.5)	33 (91.7)	20 (32.3)	4 (33.3)	2 (25.0)	5 (100)
Other^†^	0	0	1 (1.6)	0	0	0
Prior lines of therapy						
First	6 (75.0)	23 (63.9)	43 (69.4)	6 (50.0)	5 (62.5)	3 (60.0)
Second	1 (12.5)	6 (16.7)	18 (29.0)	6 (50.0)	3 (37.5)	1 (20.0)
Third	0	5 (13.9)	1 (1.6)	0	0	1 (20.0)
≥Fourth	1 (12.5)	2 (5.6)	0	0	0	0
Baseline ECOG PS, n (%)						
0	3 (37.5)	15 (41.7)	16 (25.8)	3 (25.0)	3 (37.5)	1 (20.0)
1	4 (50.0)	9 (25.0)	42 (67.7)	8 (66.7)	4 (50.0)	4 (80.0)
2	1 (12.5)	1 (2.8)	4 (6.5)	1 (8.3)	1 (12.5)	0
Missing	0	1 (2.8)	0	0	0	0
Baseline brain metastases,^‡^ n (%)	0	8 (22.2)	11 (17.7)	1 (8.3)	1 (12.5)	1 (20.0)

ECOG PS, Eastern Cooperative Oncology Group performance status; EGFR, epidermal growth factor receptor. ^†^other: One Black/African American. ^‡^Asymptomatic.****

## Discussion

This study was a combined analysis of three phase IIIb, open-label, multicenter, single-arm trials in which EGFR TKI-naïve patients with locally advanced or metastatic *EGFR*m+ NSCLC received afatinib. Patient characteristics were comparable to those previously reported in studies of EGFR TKIs used in routine clinical practice, both globally and in Asia (14–17). The patient population included subsets that are generally under-represented in clinical trials, including the elderly (38.2% aged ≥65 years; 10.7% aged ≥75 years), patients with brain metastases (19.2%), patients with ECOG PS 2 (4.4%), and those with tumors harboring uncommon *EGFR* mutations (17.9%).

In this diverse patient population, afatinib was generally tolerable with no new or unexpected safety findings. The most common AEs were EGFR TKI class-related toxicities (diarrhea, rash/acne, stomatitis, and paronychia) consistent with findings from the LUX-Lung 3, 6, and 7 studies (8, 9, 11). The overall rate of dose reductions due to AEs (41.6%) was similar to that reported in the LUX-Lung 3 and 7 studies (52% and 39%, respectively) (8, 11), but were more frequent than in LUX-Lung 6 (28%) (9), possibly reflecting differences in side effect management in different populations. However, consistent with RCT data (21, 22), and real-world studies (23), TRAEs rarely led to afatinib discontinuation in everyday clinical practice.

PFS and objective response rates (ORR) in this study are comparable to afatinib real-world studies (median PFS: 11.8–19.1 months; ORR: 67.1-76.5%) (14, 16, 17) and in the LUX-Lung trials, (median PFS 11.0–11.1 months; ORR: 56–70%) (8, 9, 11). At 14.8 months, median TTSP was almost 2 months longer than the median PFS, indicating that, following tumor progression, patients obtained clinical benefit from afatinib for another ~2 months on average, before clinically significant symptomatic progression was identified and treatment was suspended. Of note, the constituent studies in this analysis were largely undertaken before osimertinib was widely available as a second-line treatment option in patients with T790M-mediated acquired resistance to EGFR TKIs. Therefore, as it is estimated that 50–70% of patients treated with afatinib acquire the T790M mutation (24), the observation of widespread treatment beyond progression in this study probably does not reflect contemporary treatment practices, especially as tumor re-biopsies at the point of radiological progression are becoming more commonplace (25). In patients who acquire the T790M mutation, treatment with osimertinib should not be delayed. Nevertheless, in patients with *EGFR*m+ NSCLC and no obvious targeted second-line treatment options after failure of afatinib, continuing treatment beyond radiological progression could be an appropriate strategy in the absence of clinical deterioration.

Limited data are available to guide treatment choices in older patients with NSCLC, which can be complicated by age-related factors such as comorbidities and polypharmacy (26). Consistent with previous studies (26), afatinib appeared to be generally effective, and tolerable, in the elderly patients included in this analysis. Indeed, when using an age cut-off of 65 years, outcomes were actually slightly improved in older compared to younger patients, which is consistent with accumulating evidence that EGFR TKIs may actually be more effective in prolonging PFS in older patients compared with younger patients (27, 28). We found that poor performance status (ECOG PS ≥2) was associated with worse efficacy outcomes with afatinib than in patients with ECOG PS 0/1; however, this analysis was based on only 4.4% of the patient population with ECOG PS 2, therefore limiting robust analysis of these findings. These findings illustrate that chronological age alone should not determine the choice of treatment in elderly patients with NSCLC, and that biological age is more relevant for predicting treatment efficacy and safety.

Patients with *EGFR*m+ NSCLC are particularly susceptible to developing brain metastases, both at diagnosis and during the disease course (15, 29). Consistent with previous studies (18, 30), the efficacy and safety of afatinib was not affected by the presence of stable brain metastases. Other studies have indicated that afatinib can cross the blood-brain-barrier, is active against symptomatic brain metastases and mitigates the risk of CNS progression (15, 31). Overall, therefore, afatinib appears to be a treatment option in patients with CNS involvement or at risk of CNS progression.

Consistent with previous findings (32, 33), this analysis demonstrated that afatinib was effective against ‘major’ uncommon mutations (G719, L761, and S768) and compound mutations. Contrary to results demonstrated in a previous study (34), efficacy was observed with afatinib across all treatment lines, including in patients with previous chemotherapy or EGFR TKI failure. Afatinib was also active in some patients with tumors harboring exon 20 insertions or ‘other’ *EGFR* mutations; however, novel therapies including mobocertinib (35), poziotinib (36) and the recently approved amivantamab (37) have shown promising activity in early phase clinical trials in tumors harboring exon 20 insertions, and may prove to be more effective for this subgroup of patients. Nevertheless, while new effective treatment options are becoming available, it is unclear whether all exon 20 insertion mutations respond to amivantamab and other agents. More detailed data are therefore required to assess the sensitivity of individual mutations but it may be that EGFR TKIs could be an option in a subset of this highly heterogeneous group.

This broad activity reflects preclinical findings showing that many uncommon *EGFR* mutations, including compound and very rare mutations, are sensitive to afatinib (38). The finding that compound *EGFR* mutations (where an EGFR-TKI sensitizing or other mutation is identified together with a mutation of unknown clinical significance) (39) are particularly sensitive to treatment with afatinib is notable, as these mutations are identified in up to one quarter of *EGFR* mutation-positive NSCLC tumors and are associated with poor prognosis (39–41). Our findings suggest that afatinib may be considered as a treatment option if a compound mutation is detected, particularly for compound mutations that include a major mutation.

This study had several limitations. Its open-label design means that the results should be interpreted with caution, particularly regarding the impact of afatinib on survival outcomes. Additionally, next-generation sequencing was unavailable for all samples, therefore limiting the scope of analysis for known negative predictive factors such as concurrent non-*EGFR* co-mutations and the effect of allele frequency (42, 43). Furthermore, all radiological assessments and *EGFR* mutation detection were performed locally according to the methodology used at each participating institution. Finally, exploratory subgroup analyses were conducted post-hoc, meaning that no formal statistical comparisons could be conducted, thus limiting the strength of the conclusions

In summary, the safety and efficacy results from this combined analysis of three large phase IIIb studies are generally consistent with findings from subanalyses of previous RCTs and real-world studies of afatinib in *EGFR*m+ NSCLC. Afatinib was tolerable and demonstrated encouraging efficacy across different patient subgroups, including patients with brain metastases and those with tumors harboring uncommon *EGFR* mutations.

## Data Availability Statement

The raw data supporting the conclusions of this article will be made available by the authors, without undue reservation.

## Ethics Statement

All studies were approved by the Institutional Review Board or Independent Ethics Committee of each participating center, and were carried out in accordance with the Declaration of Helsinki, the International Conference on Harmonisation of Technical Requirements for Pharmaceuticals for Human Use, Good Clinical Practice, and local laws. This is not a primary study but a pooled analysis of previous studies. All patients provided written, informed consent.

## Author Contributions

APa: Formal analysis, investigation, resources, and manuscript writing. FdM: Formal analysis, investigation, resources, and manuscript writing. H-YT: Investigation and manuscript writing (review and editing). KKL: Formal analysis, investigation, resources, and manuscript writing (review and editing). JF: Investigation and manuscript writing (review and editing). APo: Formal analysis, investigation, resources, and manuscript writing (review and editing). JZ: Investigation and manuscript writing (review and editing). EHT: Investigation and manuscript writing (review and editing). MG: Manuscript writing (review and editing). VL: Conceptualization, methodology, validation, investigation, resources, and manuscript writing (original draft, review, and editing). DK: Formal analysis, investigation, data curation and manuscript writing (original draft, review, and editing). CY: Investigation and manuscript writing (review and editing). BS: Investigation and manuscript writing (review and editing). LC: Manuscript writing (review and editing). TJ: Investigation and manuscript writing (review and editing). DCLH: Investigation and manuscript writing (review and editing). AC: Conceptualization, methodology and manuscript writing (original draft, review, and editing). KP: Formal analysis, investigation, resources, and manuscript writing. Y-LW: Formal analysis, investigation, resources, and manuscript writing. All authors contributed to the article and approved the submitted version.

## Funding

This work was supported by Boehringer Ingelheim International GmbH.

## Conflict of Interest

LC is an employee of Boehringer Ingelheim Italia S.p.A. TJ is an employee of Syneos Health. DCLH is an employee of Boehringer Ingelheim Taiwan Limited. AC is an employee of Boehringer Ingelheim International GmbH.

APa received honoraria for consulting, advisory role or lectures from AstraZeneca, Agilent/Dako, Boehringer Ingelheim, Bristol-Myers Squibb, Eli Lilly, Jansenn, Merck Sharp & Dohme, Pfizer and Roche Genentech. FdM has received honoraria or consulting fees from AstraZeneca, Bristol-Myers Squibb, Merck Sharp & Dohme and Roche. KKL reports receiving advisory council or committee fees from Boehringer-Ingelheim, Bristol Myers Squibb, Merck Sharp & Dohme, Merck, Amgen, Roche, Takeda, Pfizer; and grants or funds from Boehringer Ingelheim, Bristol Myers Squibb, Merck Sharp & Dohme, Merck, Amgen, Roche, Takeda, Pfizer. VL reports receiving honoraria from AstraZeneca, Eli Lilly, Novartis, Roche, Merck Sharp & Dohme. DK reports receiving advisory council or committee fees from Boehringer Ingelheim, Bristol Myers Squibb, Merck Sharp & Dohme, Merck, Amgen, Roche-Genentech, Takeda, Pfizer; and consulting fees from Boehringer Ingelheim, Bristol Myers Squibb, Merck Sharp & Dohme, Merck, Amgen, Roche-Genentech, Pfizer. KP reports receiving personal fees from Amgen, Astellas, AstraZeneca, Boehringer Ingelheim, Clovis, Daiichi Sankyo, Eli Lilly, Hanmi, Kyowa Hakko Kirin, Incyte, LOXO, Merck KGaA, Merck Sharp & Dohme, Ono, Novartis, and Roche; and research funding from AstraZeneca and Merck Sharp & Dohme. Y-LW reports receiving honoraria from AstraZeneca, Boehringer Ingelheim, Bristol Myers Squibb, Eli Lilly, Merck Sharp & Dohme, Pfizer, Sanofi, Roche; and grants/patents received or pending from AstraZeneca, Boehringer Ingelheim, Bristol Myers Squibb.

The remaining authors declare that the research was conducted in the absence of any commercial or financial relationships that could be construed as a potential conflict of interest.

The authors declare that this study received funding from Boehringer Ingelheim International GmbH. The study sponsor participated in the design of the studies, the collection, analysis, and interpretation of the data, writing this article, and the decision to submit the article for publication.

## References

[1] Shah R Lester JF. Tyrosine Kinase Inhibitors for the Treatment of EGFR Mutation-Positive Non-Small-Cell Lung Cancer: A Clash of the Generations. Clin Lung Cancer (2020) 21:e216–e28. 10.1016/j.cllc.2019.12.003 32014348

[2] Hanna N Johnson D Temin S Baker S Jr Brahmer J Ellis PM. Systemic Therapy for Stage IV Non-Small-Cell Lung Cancer: American Society of Clinical Oncology Clinical Practice Guideline Update. J Clin Oncol (2017) 35:3484–515. 10.1200/jco.2017.74.6065 28806116

[3] Planchard D Popat S Kerr K Novello S Smit EF Faivre-Finn C. Metastatic Non-Small Cell Lung Cancer: ESMO Clinical Practice Guidelines for Diagnosis, Treatment and Follow-Up. Ann Oncol (2018) 29:iv192–237. 10.1093/annonc/mdy275 30285222

[4] Girard N. Optimizing Outcomes and Treatment Sequences in EGFR Mutation-Positive Non-Small-Cell Lung Cancer: Recent Updates. Future Oncol (2019) 15:2983–97. 10.2217/fon-2019-0400 31452384

[5] Wu YL Planchard D Lu S Sun H Yamamoto N Kim DW. Pan-Asian Adapted Clinical Practice Guidelines for the Management of Patients With Metastatic Non-Small-Cell Lung Cancer: A CSCO-ESMO Initiative Endorsed by JSMO, KSMO, MOS, SSO and TOS. Ann Oncol (2019) 30:171–210. 10.1093/annonc/mdy554 30596843

[6] Li D Ambrogio L Shimamura T Kubo S Takahashi M Chirieac LR. BIBW2992, an Irreversible EGFR/HER2 Inhibitor Highly Effective in Preclinical Lung Cancer Models. Oncogene (2008) 27:4702–11. 10.1038/onc.2008.109 PMC2748240 18408761

[7] Solca F Dahl G Zoephel A Bader G Sanderson M Klein C. Target Binding Properties and Cellular Activity of Afatinib (BIBW 2992), an Irreversible ErbB Family Blocker. J Pharmacol Exp Ther (2012) 343:342–50. 10.1124/jpet.112.197756 22888144

[8] Sequist LV Yang JC Yamamoto N O'Byrne K Hirsh V Mok T. Phase III Study of Afatinib or Cisplatin Plus Pemetrexed in Patients With Metastatic Lung Adenocarcinoma With EGFR Mutations. J Clin Oncol (2013) 31:3327–34. 10.1200/jco.2012.44.2806 23816960

[9] Wu YL Zhou C Hu CP Feng J Lu S Huang Y. Afatinib Versus Cisplatin Plus Gemcitabine for First-Line Treatment of Asian Patients With Advanced Non-Small-Cell Lung Cancer Harbouring EGFR Mutations (LUX-Lung 6): An Open-Label, Randomised Phase 3 Trial. Lancet Oncol (2014) 15:213–22. 10.1016/s1470-2045(13)70604-1 24439929

[10] Yang JC Wu YL Schuler M Sebastian M Popat S Yamamoto N. Afatinib Versus Cisplatin-Based Chemotherapy for EGFR Mutation-Positive Lung Adenocarcinoma (LUX-Lung 3 and LUX-Lung 6): Analysis of Overall Survival Data From Two Randomised, Phase 3 Trials. Lancet Oncol (2015) 16:141–51. 10.1016/s1470-2045(14)71173-8 25589191

[11] Park K Tan EH O'Byrne K Zhang L Boyer M Mok T. Afatinib Versus Gefitinib as First-Line Treatment of Patients With EGFR Mutation-Positive Non-Small-Cell Lung Cancer (LUX-Lung 7): A Phase 2B, Open-Label, Randomised Controlled Trial. Lancet Oncol (2016) 17:577–89. 10.1016/s1470-2045(16)30033-x 27083334

[12] Paz-Ares L Tan EH O'Byrne K Zhang L Hirsh V Boyer M. Afatinib Versus Gefitinib in Patients With EGFR Mutation-Positive Advanced Non-Small-Cell Lung Cancer: Overall Survival Data From the Phase IIb LUX-Lung 7 Trial. Ann Oncol (2017) 28:270–7. 10.1093/annonc/mdw611 PMC5391700 28426106

[13] Sherman RE Anderson SA Dal Pan GJ Gray GW Gross T Hunter NL. Real-World Evidence - What Is It and What Can It Tell Us? N Engl J Med (2016) 375:2293–7. 10.1056/NEJMsb1609216 27959688

[14] Liang SK Hsieh MS Lee MR Keng LT Ko JC Shih JY. Real-World Experience of Afatinib as a First-Line Therapy for Advanced EGFR Mutation-Positive Lung Adenocarcinoma. Oncotarget (2017) 8:90430–43. 10.18632/oncotarget.19563 PMC5685763 29163842

[15] Hochmair MJ Morabito A Hao D Yang CT Soo RA Yang JC. Sequential Treatment With Afatinib and Osimertinib in Patients With EGFR Mutation-Positive Non-Small-Cell Lung Cancer: An Observational Study. Future Oncol (2018) 14:2861–74. 10.2217/fon-2018-0711 30336693

[16] Ho GF Chai CS Alip A Wahid MIA Abdullah MM Foo YC. Real-World Experience of First-Line Afatinib in Patients With EGFR-Mutant Advanced NSCLC: A Multicenter Observational Study. BMC Cancer (2019) 19:896. 10.1186/s12885-019-6107-1 31500587 PMC6734518

[17] Kim Y Lee SH Ahn JS Ahn MJ Park K Sun JM. Efficacy and Safety of Afatinib for EGFR-Mutant Non-Small Cell Lung Cancer, Compared With Gefitinib or Erlotinib. Cancer Res Treat (2019) 51:502–9. 10.4143/crt.2018.117 PMC6473268 29898592

[18] Park K Wan-Teck Lim D Okamoto I Yang JC. First-Line Afatinib for the Treatment of EGFR Mutation-Positive Non-Small-Cell Lung Cancer in the 'Real-World' Clinical Setting. Ther Adv Med Oncol (2019) 11:1758835919836374. 10.1177/1758835919836374 31019567 PMC6466470

[19] Wu Y Tu H Feng J Shi M Zhao J Wang Y. P2.01-99 A Phase IIIb Open-Label Study of Afatinib in EGFR TKI-Naïve Patients With EGFR Mutation-Positive NSCLC: Final Analysis. J Thorac Oncol (2019) 14:S679–S80. 10.1016/j.jtho.2019.08.1442

[20] de Marinis F Laktionov KK Poltoratskiy A Egorova I Hochmair M Passaro A. Afatinib in EGFR TKI-Naïve Patients With Locally Advanced or Metastatic EGFR Mutation-Positive Non-Small Cell Lung Cancer: Interim Analysis of a Phase 3b Study. Lung Cancer (2020) 152:127–34. 10.1016/j.lungcan.2020.12.011 33387727

[21] Yang JC Sequist LV Zhou C Schuler M Geater SL Mok T. Effect of Dose Adjustment on the Safety and Efficacy of Afatinib for EGFR Mutation-Positive Lung Adenocarcinoma: Post Hoc Analyses of the Randomized LUX-Lung 3 and 6 Trials. Ann Oncol (2016) 27:2103–10. 10.1093/annonc/mdw322 27601237

[22] Schuler M Tan EH O'Byrne K Zhang L Boyer M Mok T. First-Line Afatinib vs Gefitinib for Patients With EGFR Mutation-Positive NSCLC (LUX-Lung 7): Impact of Afatinib Dose Adjustment and Analysis of Mode of Initial Progression for Patients Who Continued Treatment Beyond Progression. J Cancer Res Clin Oncol (2019) 145:1569–79. 10.1007/s00432-019-02862-x PMC6527523 30783814

[23] Halmos B Tan EH Soo RA Cadranel J Lee MK Foucher P. Impact of Afatinib Dose Modification on Safety and Effectiveness in Patients With EGFR Mutation-Positive Advanced NSCLC: Results From a Global Real-World Study (RealGiD). Lung Cancer (2019) 127:103–11. 10.1016/j.lungcan.2018.10.028 30642537

[24] Girard N. Optimizing Outcomes in EGFR Mutation-Positive NSCLC: Which Tyrosine Kinase Inhibitor and When? Future Oncol (2018) 14:1117–32. 10.2217/fon-2017-0636 29336166

[25] Kerr KM Bibeau F Thunnissen E Botling J Ryška A Wolf J. The Evolving Landscape of Biomarker Testing for Non-Small Cell Lung Cancer in Europe. Lung Cancer (2021) 154:161–75. 10.1016/j.lungcan.2021.02.026 33690091

[26] Wu YL Sequist LV Tan EH Geater SL Orlov S Zhang L. Afatinib as First-Line Treatment of Older Patients With EGFR Mutation-Positive Non-Small-Cell Lung Cancer: Subgroup Analyses of the LUX-Lung 3, LUX-Lung 6, and LUX-Lung 7 Trials. Clin Lung Cancer (2018) 19:e465–79. 10.1016/j.cllc.2018.03.009 29653820

[27] Imai H Kaira K Suzuki K Anzai M Tsuda T Ishizuka T. A Phase II Study of Afatinib Treatment for Elderly Patients With Previously Untreated Advanced Non-Small-Cell Lung Cancer Harboring EGFR Mutations. Lung Cancer (2018) 126:41–7. 10.1016/j.lungcan.2018.10.014 30527191

[28] Roviello G Zanotti L Cappelletti MR Gobbi A Dester M Paganini G. Are EGFR Tyrosine Kinase Inhibitors Effective in Elderly Patients With EGFR-Mutated Non-Small Cell Lung Cancer? Clin Exp Med (2018) 18:15–20. 10.1007/s10238-017-0460-7 28391544

[29] Li L Luo S Lin H Yang H Chen H Liao Z. Correlation Between EGFR Mutation Status and the Incidence of Brain Metastases in Patients With Non-Small Cell Lung Cancer. J Thorac Dis (2017) 9:2510–20. 10.21037/jtd.2017.07.57 PMC5594201 28932557

[30] Schuler M Wu YL Hirsh V O'Byrne K Yamamoto N Mok T. First-Line Afatinib Versus Chemotherapy in Patients With Non-Small Cell Lung Cancer and Common Epidermal Growth Factor Receptor Gene Mutations and Brain Metastases. J Thorac Oncol (2016) 11:380–90. 10.1016/j.jtho.2015.11.014 26823294

[31] Hochmair M Holzer S Burghuber OC. Complete Remissions in Afatinib-Treated Non-Small-Cell Lung Cancer Patients With Symptomatic Brain Metastases. Anticancer Drugs (2016) 27:914–5. 10.1097/cad.0000000000000410 27442131

[32] Passaro A Mok T Peters S Popat S Ahn MJ de Marinis F. Recent Advances on the Role of EGFR Tyrosine Kinase Inhibitors in the Management of NSCLC With Uncommon, Non Exon 20 Insertions, EGFR Mutations. J Thorac Oncol (2021) 16:764–73. 10.1016/j.jtho.2020.12.002 33333327

[33] Yang JC Schuler M Popat S Miura S Heeke S Park K. Afatinib for the Treatment of NSCLC Harboring Uncommon EGFR Mutations: A Database of 693 Cases. J Thorac Oncol (2020) 15:803–15. 10.1016/j.jtho.2019.12.126 31931137

[34] Landi L Tiseo M Chiari R Ricciardi S Rossi E Galetta D. Activity of the EGFR-HER2 Dual Inhibitor Afatinib in EGFR-Mutant Lung Cancer Patients With Acquired Resistance to Reversible EGFR Tyrosine Kinase Inhibitors. Clin Lung Cancer (2014) 15:411–7.e4. 10.1016/j.cllc.2014.07.002 25242668

[35] Janne PA Neal JW Camidge DR Spira AI Piotrowska Z Horn L. Antitumor Activity of TAK-788 in NSCLC With EGFR Exon 20 Insertions. J Clin Oncol (2019) 37:9007. 10.1200/JCO.2019.37.15_suppl.9007

[36] Sacher A Le X Cornelissen R Shum E Suga J Socinski M. 36MO Safety, Tolerability and Preliminary Efficacy of Poziotinib With Twice Daily Strategy in EGFR/HER2 Exon 20 Mutant Non-Small Cell Lung Cancer. Ann Oncol (2021) 32:S15. 10.1016/j.annonc.2021.01.051

[37] Sabari JK Shu CA Park K Leighl N Mitchell PL Kim S-w. (2020). OA04.04 Amivantamab in Post-Platinum EGFR Exon 20 Insertion Mutant Non-Small Cell Lung Cancer. J Thorac Oncol (2021) 16(Suppl3):S108–9. 10.1016/j.jtho.2021.01.284

[38] Kohsaka S Nagano M Ueno T Suehara Y Hayashi T Shimada N. A Method of High-Throughput Functional Evaluation of EGFR Gene Variants of Unknown Significance in Cancer. Sci Transl Med (2017) 9:eaan6566. 10.1126/scitranslmed.aan6566 29141884

[39] Kobayashi S Canepa HM Bailey AS Nakayama S Yamaguchi N Goldstein MA. Compound EGFR Mutations and Response to EGFR Tyrosine Kinase Inhibitors. J Thorac Oncol (2013) 8:45–51. 10.1097/JTO.0b013e3182781e35 PMC3531043 23242437

[40] Kim EY Cho EN Park HS Hong JY Lim S Youn JP. Compound EGFR Mutation Is Frequently Detected With Co-Mutations of Actionable Genes and Associated With Poor Clinical Outcome in Lung Adenocarcinoma. Cancer Biol Ther (2016) 17:237–45. 10.1080/15384047.2016.1139235 PMC4848002 26785607

[41] Kohsaka S Petronczki M Solca F Maemondo M. Tumor Clonality and Resistance Mechanisms in EGFR Mutation-Positive Non-Small-Cell Lung Cancer: Implications for Therapeutic Sequencing. Future Oncol (2019) 15:637–52. 10.2217/fon-2018-0736 30404555

[42] Chevallier M Tsantoulis P Addeo A Friedlaender A. Influence of Concurrent Mutations on Overall Survival in EGFR-Mutated Non-Small Cell Lung Cancer. Cancer Genom Proteom (2020) 17:597–603. 10.21873/cgp.20216 PMC7472449 32859638

[43] Friedlaender A Tsantoulis P Chevallier M De Vito C Addeo A. The Impact of Variant Allele Frequency in EGFR Mutated NSCLC Patients on Targeted Therapy. Front Oncol (2021) 11:644472. 10.3389/fonc.2021.644472 33869038 PMC8044828

