# Supplementary material for: Afatinib in EGFR TKI-Naïve Patients with Locally Advanced or Metastatic EGFR Mutation-Positive Non-Small Cell Lung Cancer: A Pooled Analysis of Three Phase IIIb Studies
Source: Front Oncol. 2021 Jul 9;11:709877. doi: 10.3389/fonc.2021.709877 (PMC8298067; doi:10.3389/fonc.2021.709877)
Supplement: Supplementary file 1 [file DataSheet_1.docx]

**Afatinib in EGFR TKI-Naïve Patients with Locally Advanced or Metastatic *EGFR* Mutation-Positive Non-Small Cell Lung Cancer: A Pooled Analysis of Three Phase IIIb Studies**

Antonio Passaro, et al.

| **Category** | **Patient subgroup** | | | |
| --- | --- | --- | --- | --- |
| All patients |  | | | |
| N | 1108 | | | |
| Median DOR, months (95% CI) | 13.2 (12.2–14.4) | | | |
| Median DDC, months (95% CI) | 14.1 (13.6–14.8) | | | |
| *EGFR* mutation type^†^ | Common^†^ | | Uncommon^‡^ | |
| N | 909 | | 198 | |
| Median DOR, months (95% CI) | 13.6 (12.5–14.8) | | 10.2 (8.4–12.9) | |
| Median DDC, months (95% CI) | 14.7 (13.9–15.9) | | 9.1 (8.1–11.4) | |
| ECOG PS | 0/1 | | 2 | |
| N | 1058 | | 49 | |
| Median DOR, months (95% CI) | 13.2 (12.2–14.8) | | 8.3 (5.0–13.0) | |
| Median DDC, months (95% CI) | 14.3 (13.7–15.2) | | 9.9 (7.4–13.9) | |
| ECOG PS (patients with common mutations)^†^ | 0/1 | | 2 | |
| N | 869 | | 40 | |
| Median DOR, months (95% CI) | 13.8 (12.5–14.9) | | 12.4 (5.0–14.1) | |
| Median DDC, months (95% CI) | 14.8 (14.1–16.3) | | 9.9 (6.2–13.9) | |
| Afatinib line of therapy | First-line | Second-line | | >Second-line |
| N | 770 | 261 | | 77 |
| Median DOR, months (95% CI) | 13.4 (12.2–14.8) | 12.6 (11.1–16.6) | | 12.5 (8.3–18.7) |
| Median DDC, months (95% CI) | 14.5 (13.7–15.9) | 13.7 (12.9–15.7) | | 12.0 (8.3–14.5) |
| Brain metastases at screening^§^ | Yes | | No | |
| N | 213 | | 894 | |
| Median DOR, months (95% CI) | 11.1 (8.3–12.3) | | 14.1 (13.0–15.4) | |
| Median DDC, months (95% CI) | 11.7 (10.2–13.8) | | 14.7 (13.9–16.1) | |
| Age, years | <75 years | | ≥75 years | |
| N | 989 | | 119 | |
| Median DOR, months (95% CI) | 13.0 (12.0–14.1) | | 16.5 (10.3–21.2) | |
| Median DDC, months (95% CI) | 14.1 (13.6–15.0) | | 14.1 (12.6–21.4) | |
| Age, years | <65 years | | ≥65 years | |
| N | 685 | | 423 | |
| Median DOR, months (95% CI) | 12.5 (11.3–13.8) | | 14.2 (12.4–17.3) | |
| Median DDC, months (95% CI) | 13.7 (12.8–14.5) | | 15.0 (13.8–17.3) | |

**SUPPLEMENTARY TABLE 1 |** Post-hoc analysis of duration of objective response and duration of disease control for specified subgroups.

*CI, confidence interval; DDC, duration of disease control; DOR, duration of objective response; ECOG PS, Eastern Cooperative Oncology Group performance status; EGFR, epidermal growth factor receptor.* ***^†^****Patients with EGFR mutation categories of Del19 only or L858R only. ^‡^Patients with EGFR mutation categories other than Exon19 only and L858R only. ^§^Asymptomatic.*

|  | **T790M  (n = 8)** | **Exon 20 ins  (n = 36)** | **Major  (n = 62)** | **Compound  (n = 12)** | **Compound with Major  (n = 8)** | **Other  (n = 5)** |
| --- | --- | --- | --- | --- | --- | --- |
| Patients with objective response, n (%) | 1 (12.5) | 9 (25.0) | 28 (45.2) | 6 (50.0) | 4 (50.0) | 2 (40.0) |
| Median DOR, months (95% CI) | 1.1 (NE–NE) | 9.1 (5.6–21.2) | 11.1 (7.1–17.2) | 17.9 (10.2–35.3) | 17.9 (10.2–35.3) | 4.7 (2.7–6.8) |

**SUPPLEMENTARY TABLE 2 |** Objective response rate and duration of objective response in patients with uncommon *EGFR* mutations.

*CI, confidence interval; DOR, duration of objective response; NE, not evaluable.*

**SUPPLEMENTARY FIGURE 1 |** Patient disposition. AEs, adverse events.
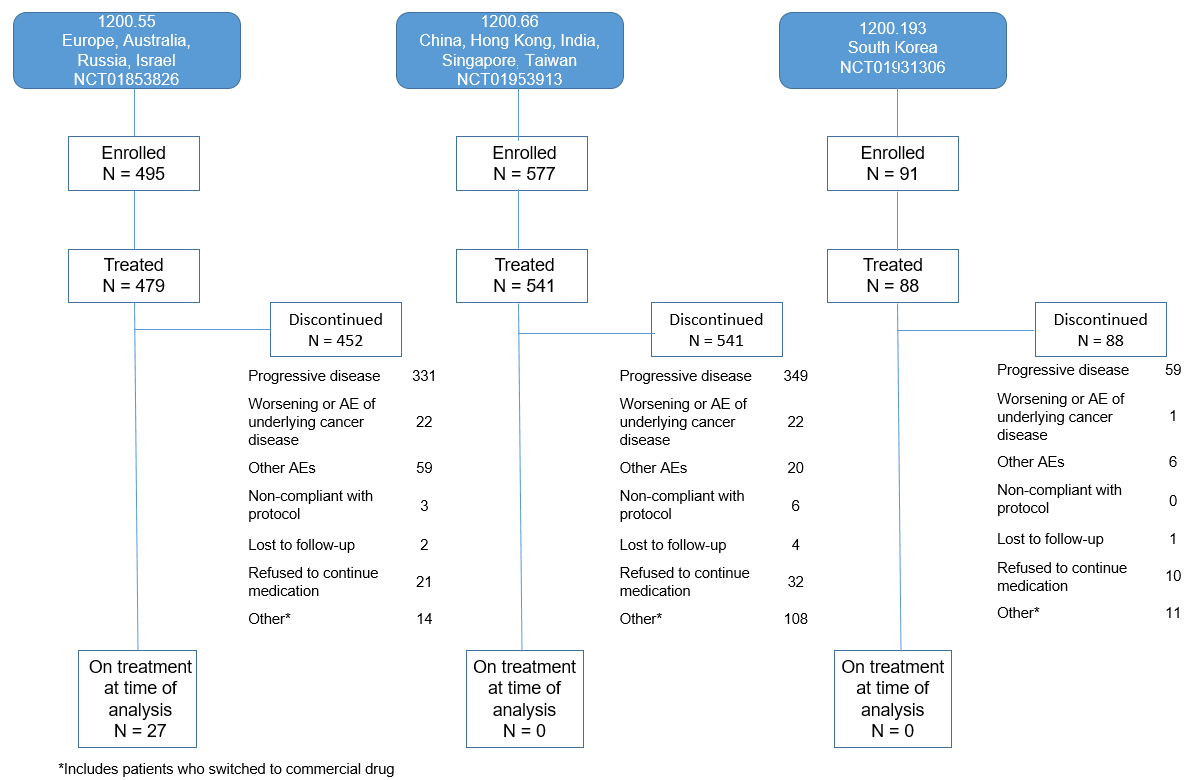


**SUPPLEMENTARY FIGURE 2** | TTSP in **(A)** all patients, **(B)** patients with tumors harboring common versus uncommon mutations, **(C)** patients with ECOG PS 0/1 versus 2, **(D)** patients with common mutations and ECOG PS 0/1 versus 2, **(E)** patients with vs without baseline brain metastases, **(F)** patients treated with afatinib in first, second and later lines of therapy, **(G)** patients aged <65 or ≥65 years, and **(H)** patients aged <75 or ≥75 years. Abbreviations: CI, confidence interval; ECOG PS, Eastern Cooperative Oncology Group performance status; TTSP, time to symptomatic progression.

**
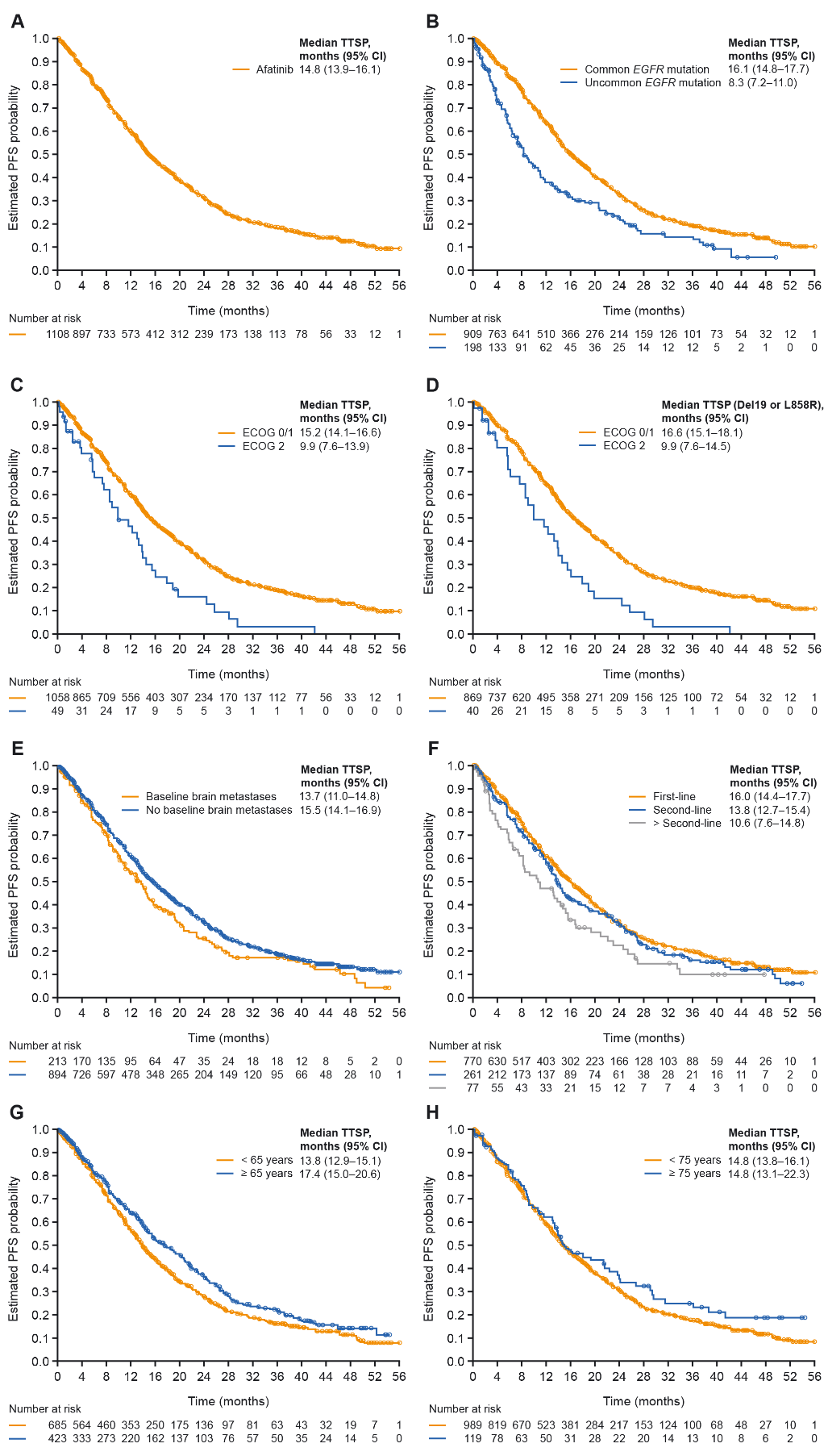
**
